# Comparative Phytochemical Profiles and Antioxidant and Tyrosinase-Inhibitory Activities of Wild and In Vitro-Derived *Curcuma lampangensis* Saensouk, Maknoi & Rakarcha

**DOI:** 10.3390/plants15172713

**Published:** 2026-09-04

**Authors:** Anchalee Phoothonrat, Piyaporn Saensouk, Surapon Saensouk, Sarayut Rakarcha, Suthira Maneechai

**Affiliations:** 1 Department of Biology, Faculty of Science, Mahasarakham University, Maha Sarakham 44150, Thailand; anchaleephoothonrat@gmail.com (A.P.); pcornukaempferia@yahoo.com (P.S.); 2 Walai Rukhavej Botanical Research Institute, Mahasarakham University, Maha Sarakham 44150, Thailand; surapon.s@msu.ac.th; 3 Diversity of Family Zingiberaceae and Vascular Plant for Its Applications Research Unit, Mahasarakham University, Maha Sarakham 44150, Thailand; 4 Sirikit Botanic Garden, The Botanical Garden Organization, Chiang Mai 50180, Thailand; sarayut.rakarcha@gmail.com

**Keywords:** *Curcuma lampangensis*, antioxidant activity, tyrosinase inhibition, phytochemical profile, in vitro culture, HPLC, LC–MS/MS, conservation

## Abstract

*Curcuma lampangensis*, a critically endangered species endemic to Thailand, has been successfully propagated in vitro; however, its phytochemical profile and biological activities remain uncharacterized. This study compared eight ethanol and ethyl acetate extracts from the aerial and belowground tissues of wild and in vitro-derived plants. Total phenolic and flavonoid contents, DPPH and ABTS radical-scavenging activities, and tyrosinase-inhibitory activity were evaluated alongside chemical profiling by HPLC-DAD, targeted LC–MS/MS, and GC–MS. The ethyl acetate extract of wild belowground tissues showed the highest TPC (5.21 mg GAE/g DW), while the ethyl acetate extract of in vitro-derived aerial tissues exhibited the highest TFC (1.59 mg QE/g DW) and ABTS activity (63.11%). The ethanol extract of wild belowground tissues showed the strongest DPPH activity (70.48%), and the ethyl acetate extract of in vitro-derived belowground tissues demonstrated the greatest tyrosinase inhibition (52.12%). HPLC and LC–MS/MS revealed a diverse phenolic and flavonoid profile, with p-coumaric acid, 4-hydroxybenzoic acid, chlorogenic acid, and quercetin consistently detected across all extracts, while ferulic and caffeic acids were widely distributed. GC–MS tentatively identified ethyl palmitate, linoleic acid ethyl ester, and ethyl oleate as predominant constituents. These findings demonstrate that in vitro-derived biomass retains appreciable phytochemical and biological properties, supporting its use as a renewable, conservation-compatible material for further characterization and bioactivity-guided research.

## 1. Introduction

The Zingiberaceae, a family of perennial herbs within the order Zingiberales, comprises approximately 1600 species in 58 genera, with its greatest diversity occurring in tropical and subtropical Asia [1,2]. Many members of this family produce aromatic essential oils and specialized metabolites that accumulate predominantly in their rhizomes and other vegetative organs. In Thailand, Zingiberaceae species are economically and culturally important as foods, spices, traditional medicines, and ornamental plants. Several widely cultivated species are also recognized as rich sources of biologically active compounds. For example, ginger (*Zingiber officinale* Roscoe) contains phenolic compounds, terpenoids, flavonoids, and other antioxidant constituents, whereas turmeric (*Curcuma longa* L.) produces curcuminoids and volatile constituents, including curcumin, demethoxycurcumin, bisdemethoxycurcumin, and ar-turmerone. These compounds have been associated with antioxidant, anti-inflammatory, antimicrobial, and enzyme-inhibitory activities [3,4,5,6,7,8,9,10]. Consequently, the Zingiberaceae represent an important plant group for phytochemical, pharmacological, and conservation-oriented research.

The genus *Curcuma* includes numerous species valued for their medicinal, culinary, and ornamental properties. Among them, *Curcuma lampangensis* Saensouk, Maknoi & Rakarcha is a perennial herb endemic to Lampang Province in northern Thailand. Owing to its restricted geographical distribution and limited natural populations, the species has been provisionally assessed as critically endangered. Its narrow distribution increases its vulnerability to habitat disturbance, overcollection, and other environmental pressures. To support ex situ conservation and provide plant material without further pressure on natural populations, an in vitro propagation system has recently been developed for this species [11,12]. Such propagation systems can generate renewable plant biomass and may facilitate phytochemical and biological investigations of rare species. Nevertheless, in vitro culture conditions can alter the accumulation of secondary metabolites through changes in nutrient availability, plant growth regulators, developmental stage, and environmental conditions. Therefore, the chemical and biological equivalence of in vitro-derived tissues to those obtained from wild plants cannot be assumed.

Comparative studies of wild and in vitro-derived materials have been conducted in several Zingiberaceae species, including *Curcuma amada* Roxb., *C. longa*, and *C. larsenii* Maknoi & Jenjitt. These investigations have demonstrated that plant origin, tissue type, culture conditions, and extraction solvent may substantially influence phenolic and flavonoid contents, antioxidant capacity, and metabolite composition [13,14,15,16,17]. However, comparable information is not yet available for *C. lampangensis*. In particular, it remains unclear whether aerial and belowground tissues produced under in vitro conditions retain phytochemical characteristics and biological activities comparable to those of wild plants. Addressing this question is important for determining whether in vitro-derived biomass can be used as a conservation-compatible substitute for wild-collected material in subsequent phytochemical investigations.

Tyrosinase is a copper-containing enzyme involved in melanin biosynthesis and enzymatic browning. Excessive tyrosinase activity is associated with hyperpigmentation, whereas enzymatic browning contributes to quality deterioration in agricultural and food products. Consequently, naturally derived tyrosinase inhibitors are of interest in pharmaceutical, cosmetic, and food-related applications. Compounds such as kojic acid, arbutin, ascorbic acid, and quercetin are commonly used as reference inhibitors, although limitations related to stability, efficacy, or safety have encouraged continued investigation of plant-derived alternatives. Tyrosinase-inhibitory activity has been reported in several Zingiberaceae taxa, including species of *Etlingera*, *Curcuma*, *Kaempferia* and *Zingiber* [18,19,20,21]. Nevertheless, the tyrosinase-inhibitory potential of wild and in vitro-derived tissues of *C. lampangensis* has not been systematically compared.

Accordingly, this study aimed to compare the phytochemical composition and in vitro biological activities of ethanol and ethyl acetate extracts prepared from aerial and belowground tissues of wild and in vitro-derived *C. lampangensis*. Ethanol and ethyl acetate were selected because they differ substantially in polarity and therefore recover partially overlapping but distinct groups of phytochemicals. Ethanol, a polar protic solvent, is effective for extracting a broad range of polar-to-moderately-polar phenolic and flavonoid constituents, whereas ethyl acetate, a semi-polar solvent, can preferentially recover less-polar phenolic and other moderately lipophilic constituents. Previous studies in *Curcuma* species have demonstrated that extraction solvent markedly influences the recovery of phenolic and flavonoid constituents and the resulting antioxidant activity [22,23,24]. Therefore, ethanol and ethyl acetate were selected in the present study to broaden the chemical coverage of *C. lampangensis* compared with the use of a single extraction solvent. Total phenolic and flavonoid contents were determined, antioxidant activities were evaluated using DPPH and ABTS radical-scavenging assays, and tyrosinase-inhibitory activity was assessed. Chemical constituents were further characterized using HPLC-DAD, targeted LC–MS/MS, and GC–MS. We hypothesized that plant-material origin, tissue type, and extraction solvent would differentially influence the phytochemical composition and associated antioxidant and tyrosinase-inhibitory activities of *C. lampangensis*. The results provide baseline phytochemical information for this critically endangered species and evaluate the suitability of in vitro-derived biomass as a renewable, conservation-compatible material for compound isolation and bioactivity-guided research.

## 2. Results

### 2.1. Total Phenolic and Total Flavonoid Contents

The total phenolic content (TPC) of the eight *C. lampangensis* extracts ranged from 0.75 to 5.21 mg GAE/g DW, whereas the total flavonoid content (TFC) ranged from 0.27 to 1.59 mg QE/g DW (Table 1). Among the tested extracts, the ethyl acetate extract of wild belowground tissues exhibited the highest TPC (5.21 ± 0.01 mg GAE/g DW), followed by the corresponding ethanol extract (4.05 ± 0.02 mg GAE/g DW). By contrast, the ethyl acetate extract of in vitro-derived aerial tissues showed the highest TFC (1.59 ± 0.04 mg QE/g DW), followed by the ethyl acetate extract of wild belowground tissues (1.19 ± 0.02 mg QE/g DW). These results indicated distinct variation in phenolic and flavonoid contents among plant origins, tissue types, and extraction solvents.

### 2.2. DPPH and ABTS Radical-Scavenging Activities

The DPPH radical-scavenging activity of extracts from wild plants ranged from 16.58% to 70.48%, whereas that of extracts from in vitro-derived plants ranged from 19.04% to 35.89% (Table 2). The ethanol extract of wild belowground tissues exhibited the highest DPPH radical-scavenging activity (70.48%). Its reported IC_50_ value was 0.0154 mg/mL.

For the ABTS assay, the ethyl acetate extract of in vitro-derived aerial tissues exhibited the highest radical-scavenging activity (63.11%), with a reported IC_50_ value of 0.01829 mg/mL. The ethanol extract of wild belowground tissues showed the second-highest ABTS activity (59.46%), with a reported IC_50_ value of 0.0188 mg/mL.

### 2.3. Tyrosinase-Inhibitory Activity

Tyrosinase inhibition by extracts from wild plants ranged from 43.91% to 49.29%, whereas extracts from in vitro-derived plants exhibited inhibition values ranging from 42.49% to 52.12% at the tested concentration (Table 2). Among all extracts, the ethyl acetate extract of in vitro-derived belowground tissues showed the highest inhibitory activity (52.12%) and an IC_50_ value of 0.021 mg/mL.

To further assess the potential contribution of the phenolic compounds identified by HPLC to the observed tyrosinase-inhibitory activity, the corresponding HPLC reference standards were individually evaluated at a final concentration of 100 µM. Most standards showed no detectable inhibitory activity, while syringic acid and chlorogenic acid exhibited only weak inhibition of 4.30 ± 2.03% and 7.26 ± 3.72%, respectively. In contrast, kojic acid, used as the reference inhibitor, showed 56.72 ± 3.64% inhibition, whereas quercetin exhibited the strongest activity, with 97.58 ± 1.78% inhibition after correction for absorbance interference associated with its intrinsic color and auto-oxidation. Because quercetin and kojic acid exhibited greater than 50% inhibition, both were further subjected to IC_50_ determination. Quercetin showed an IC_50_ value of 42.23 µM (0.0128 mg/mL), while kojic acid showed an IC_50_ value of 87.93 µM (0.0125 mg/mL).

### 2.4. HPLC Profiles of Phenolic Acids and Flavonoids

**Figure 1:**
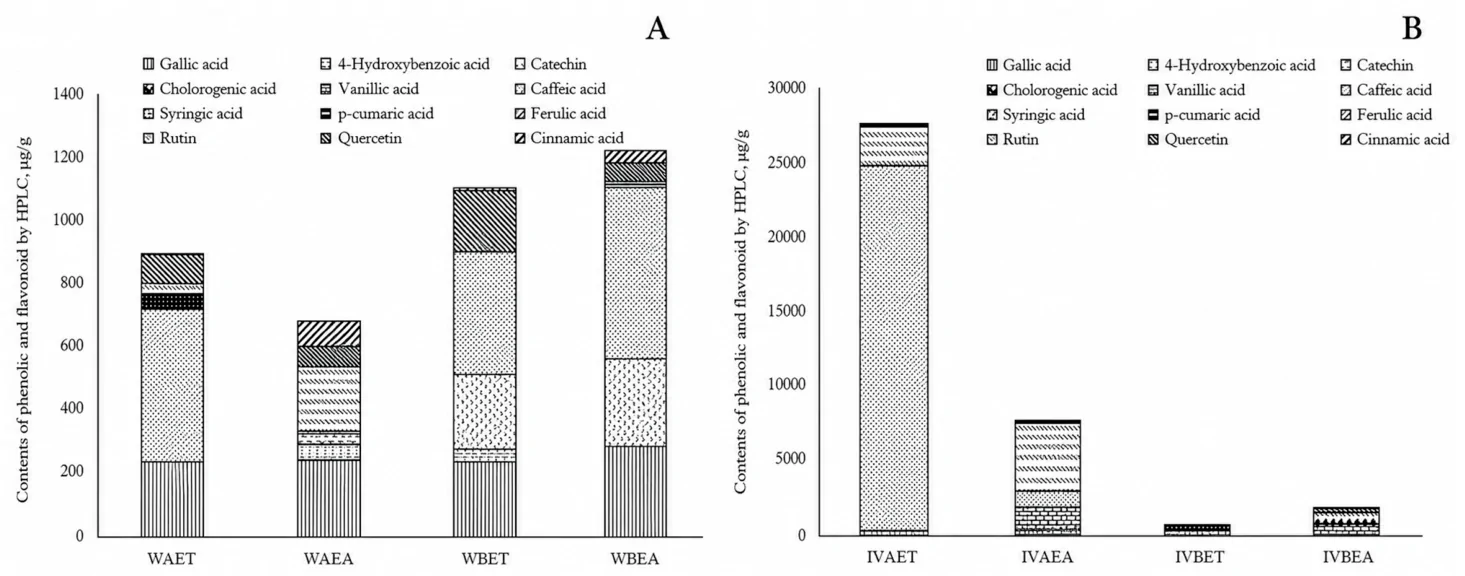
HPLC profiles of phenolic and flavonoid compounds detected in extracts *of C. lampangensis*. Stacked bars show the contents of individual compounds in (**A**) wild plant extracts and (**B**) in vitro-derived plant extracts.

HPLC analysis revealed marked qualitative and quantitative variation among extracts from different plant origins, tissue types, and extraction solvents (Figure 1). HPLC-DAD peaks corresponding to gallic acid, quercetin, and cinnamic acid were observed across the eight extracts based on comparison with authentic standards. However, subsequent targeted LC–MS/MS supported the assignment of quercetin but did not detect gallic acid or cinnamic acid under the LC–MS/MS conditions used. In contrast, several compounds showed extract-specific distributions. Caffeic acid was detected only in extracts of wild belowground tissues, whereas vanillic acid occurred predominantly in the ethyl acetate extract of in vitro-derived aerial tissues. Ferulic acid was detected mainly in in vitro-derived samples, and rutin accumulated at comparatively high levels in extracts of in vitro-derived aerial tissues.

### 2.5. Targeted LC–MS/MS Analysis of Phenolic and Flavonoid Compounds

LC–MS/MS analysis was additionally performed to strengthen the identification of phenolic and flavonoid compounds detected by HPLC. Compound identification was supported by comparison of retention times and compound-specific MRM transitions with those of authentic reference standards analyzed under identical conditions (Table 3).

Targeted LC–MS/MS analysis was performed using the same 12 reference compounds employed for HPLC identification to provide additional evidence for the occurrence of selected phenolic and flavonoid constituents in wild and in vitro-derived extracts of *C. lampangensis*. The LC–MS/MS responses varied among compounds and extract types. Among the analyzed compounds, p-coumaric acid, 4-hydroxybenzoic acid, chlorogenic acid, and quercetin were detected in all eight extracts, and targeted LC–MS/MS further supported the assignment of quercetin. Ferulic acid was detected in seven extracts, with no detectable signal in WAET, whereas caffeic acid was detected in all extracts except IVBEA. Rutin was detected in WAET, IVAET, and IVAEA, while vanillic acid was detected only in WAEA and IVAEA. Syringic acid was detected exclusively in IVAEA. In contrast, gallic acid, cinnamic acid, and catechin were not detected in any extract under the present LC–MS/MS conditions.

### 2.6. GC–MS Profiles

GC–MS analysis tentatively identified ten compounds in the ethanol and ethyl acetate extracts of wild and in vitro-derived *C. lampangensis* tissues (Table 4). Considerable variation in the relative chemical profiles was observed according to plant source, tissue type, and extraction solvent. The total relative peak areas of the retained compounds were highest in the in vitro-derived aerial ethyl acetate extract (IVAEA; 76.10%), followed by the in vitro-derived aerial ethanol extract (IVAET; 74.73%), wild belowground ethyl acetate extract (WBEA; 67.89%), and wild belowground ethanol extract (WBET; 67.17%). By contrast, only small proportions of the identified compounds were detected in the wild aerial extracts, with total relative peak areas of 1.81% in WAET and 1.56% in WAEA. No retained compound was detected in IVBET.

Fatty acid ethyl esters were the predominant constituents of the wild belowground and in vitro-derived aerial extracts. Ethyl palmitate was the most abundant compound in WBET, WBEA, and IVAET, accounting for 19.81%, 25.80%, and 34.14% of the total peak area, respectively. In IVAEA, ethyl palmitate was also abundant at 21.19%, together with ethyl linoleate (19.50%) and ethyl oleate (15.73%). Ethyl linoleate ranged from 12.71% to 19.54% among WBET, WBEA, IVAET, and IVAEA, whereas ethyl stearate was detected in WBET (8.81%), WBEA (7.47%), and IVAEA (6.89%). The combined relative peak areas of fatty acid ethyl esters were highest in IVAEA (63.31%), followed by IVAET (55.57%), WBEA (51.25%), and WBET (50.92%).

Several compounds showed source- or tissue-specific distributions. Camphor was detected exclusively in WBET at 1.46%, whereas coumarin was detected in WAET (0.83%), WAEA (1.02%), and IVAET, with its highest relative abundance recorded in IVAET at 5.75%. Hexadecane and octadecane were present in both wild belowground and in vitro-derived aerial extracts, with octadecane reaching its highest level in WBEA at 6.90%. Manool was uniquely detected in the in vitro-derived belowground ethyl acetate extract (IVBEA), where it represented 36.40% of the total peak area. Overall, the results demonstrate marked differences in the GC–MS chemical profiles of wild and in vitro-derived tissues, with the wild belowground and in vitro-derived aerial extracts being characterized predominantly by fatty acid ethyl esters.

## 3. Discussion

The comparative analysis of wild and in vitro-derived *C. lampangensis* tissues demonstrated that tissue composition, plant origin, and extraction solvent were associated with marked differences in phytochemical recovery and bioactivity. Among the extracts examined, ethyl acetate extraction of wild belowground tissue yielded the highest total phenolic content, whereas the same solvent applied to in vitro-derived aerial tissue produced the highest total flavonoid content and the most potent ABTS radical scavenging activity. A different pattern emerged for DPPH scavenging capacity, which was strongest in the ethanolic extract of wild belowground tissue, while tyrosinase inhibitory activity was most pronounced in the ethyl acetate extract of in vitro-derived belowground tissue. These findings indicate that tissue origin and solvent polarity are critical determinants of extraction efficiency for bioactive constituents, and further support the potential of in vitro-derived biomass as a sustainable alternative source of phytochemicals, notwithstanding compositional differences from wild-collected material.

### 3.1. Phenolic and Flavonoid Contents and Antioxidant Activities

The comparatively high total phenolic content observed in the wild belowground ethyl acetate extract may reflect organ-dependent accumulation of specialized metabolites in *C. lampangensis*. Belowground organs can serve as storage and perennating structures and may accumulate metabolites associated with plant defense and adaptation to environmental stress. However, the distribution of phenolic constituents between aerial and belowground organs vary considerably among members of the Zingiberaceae. In *Curcuma longa*, total phenolic content was higher in rhizomes than in leaves, whereas the opposite pattern was observed in *Etlingera maingayi*, with substantially greater phenolic content in the leaves [19]. In *Zingiber spectabile*, however, phenolic contents of leaves and rhizomes were not significantly different [19]. Higher phenolic contents in leaves than in rhizomes have also been reported for *Curcuma amada*, *Etlingera philippinensis*, and wild *C. larsenii* [16,25,26]. Collectively, these contrasting findings indicate that phenolic accumulation in Zingiberaceae is species- and organ-dependent and may be further influenced by developmental stage, growth conditions, and extraction procedures. Belowground organs can serve as storage and perennating structures and may accumulate specialized metabolites involved in defense against belowground herbivores and other environmental challenges [27,28].

The high flavonoid content and ABTS activity of the in vitro-derived aerial ethyl acetate extract are particularly noteworthy. In vitro culture can alter specialized-metabolite accumulation through controlled nutrient availability, exogenous plant growth regulators, light conditions, and developmental status. The MS medium used in the present propagation system was supplemented with 2 mg/L BA and 0.5 mg/L NAA, and exposure to these regulators may have influenced metabolic allocation in the developing tissues. Nevertheless, the current experimental design does not allow the observed differences to be attributed specifically to BA, NAA, or in vitro culture because the wild and cultured materials differed simultaneously in growth environment, developmental stage, and anatomical composition. The increased flavonoid content in in vitro-derived aerial tissues should therefore be interpreted as a culture-associated pattern rather than direct evidence of growth-regulator-induced stimulation.

Previous investigations have reported substantial variation in the phenolic contents and antioxidant activities of *Curcuma* species. For example, phenolic and flavonoid compounds have been reported in the rhizomes of *C. longa*, *C. amada*, *C. caesia*, and *C. xanthorrhiza* [29,30,31,32]. Behera et al. [13] found similar total phenolic and flavonoid contents in naturally obtained and acclimatized rhizomes of *C. amada*, whereas Saensouk et al. [33] observed organ- and culture-dependent variation in *K. larsenii*. Direct numerical comparison among these studies should, however, be made cautiously. Reported values were expressed using different bases, including dry plant weight and dry extract weight, and were generated using different solvents, extraction methods, assay conditions, and calibration standards. Consequently, differences among published values cannot be attributed solely to species identity or cultivation system.

The contrasting DPPH and ABTS responses observed among the extracts may reflect differences in the chemical principles underlying the two assays. Although both methods assess radical-scavenging capacity, DPPH is generally more responsive to compounds soluble in relatively nonpolar organic media, whereas ABTS can detect a broader range of hydrophilic and lipophilic antioxidants. Thus, the strongest DPPH activity in the wild belowground ethanol extract and the strongest ABTS activity in the in vitro-derived aerial ethyl acetate extract are not necessarily contradictory. Instead, these findings suggest that the extracts contained different mixtures of antioxidant compounds with differing polarity, reaction kinetics, and radical-scavenging mechanisms. The relatively high ABTS activity of the in vitro-derived aerial extract was accompanied by its high flavonoid content, suggesting a possible contribution of flavonoids to this activity. Nevertheless, this relationship should be verified using correlation analysis and bioactivity guided fractionation because total flavonoid content alone cannot establish a causal association.

### 3.2. Tyrosinase-Inhibitory Activity

The ethyl acetate extract of in vitro-derived belowground tissues exhibited the greatest tyrosinase inhibition among the tested extracts. This finding suggests that in vitro-derived material contains constituents capable of interfering with tyrosinase activity or substrate-mediated oxidation. Tyrosinase-inhibitory activity has previously been reported in several Zingiberaceae species, including *Etlingera elatior*, *E. fulgens*, *E. maingayi*, *E. rubrostriata*, *E. littoralis*, *Curcuma longa*, *C. heyneana*, and *Kaempferia galanga* [19]. Phenolic compounds, particularly flavonoids such as quercetin, have been associated with tyrosinase inhibition through interactions with the enzyme active site and copper ions.

To further examine the possible contribution of the phenolic compounds detected by HPLC, the corresponding reference standards were evaluated using the same tyrosinase assay. At a final concentration of 100 µM, most HPLC reference standards showed no detectable inhibitory activity, whereas syringic acid and chlorogenic acid exhibited only weak inhibition.

Quercetin exhibited strong tyrosinase-inhibitory activity, with an IC_50_ value of 42.23 µM (0.0128 mg/mL), compared with 87.93 µM (0.0125 mg/mL) for kojic acid. On a molar basis, quercetin therefore showed approximately two-fold greater inhibitory potency than kojic acid under the present assay conditions. This finding is consistent with previous studies reporting pronounced tyrosinase inhibition by quercetin. Choi et al. reported IC_50_ values of 14.31 ± 3.93 µM for quercetin and 11.38 ± 4.16 µM for kojic acid, indicating comparable inhibitory potency under the same assay conditions [34]. Jakimiuk et al. reported an IC_50_ value of 44.38 ± 0.13 µM for quercetin, which closely agrees with the value obtained in the present study, and demonstrated competitive inhibition of mushroom tyrosinase [35]. Fan et al. also reported potent tyrosinase inhibition by quercetin and suggested that its activity is associated with interactions of the 3′,4′-dihydroxy moiety with copper ions at the enzyme active site [36]. Nevertheless, because quercetin may also behave as a tyrosinase substrate or reducing agent in some assay systems, the present results are most appropriately interpreted as tyrosinase-inhibitory activity under the specific L-DOPA assay conditions employed. These results support quercetin as a potentially important contributor to the tyrosinase-inhibitory activity observed in *C. lampangensis* extracts. However, because most other HPLC-identified phenolic standards showed little or no inhibitory activity, the overall activity of the crude extracts is unlikely to be attributable to all detected phenolics equally and may instead reflect the contribution of specific active constituents, their relative abundance, and possible interactions among multiple compounds.

The IC_50_ value obtained for the in vitro-derived belowground ethyl acetate extract should nevertheless be interpreted cautiously when compared with values reported for *C. heyneana* and *K. galanga*. Previous studies have examined essential oils or crude solvent extracts using different enzyme sources, substrates, incubation conditions, and calculation procedures, all of which can substantially influence the resulting inhibitory values. In addition, direct comparison between crude extracts and isolated reference compounds should be made with caution because of differences in chemical composition and concentration expression. Taken together, the present findings support further investigation of *C. lampangensis* as a potential source of tyrosinase-inhibitory constituents and indicate that quercetin may be one of the compounds contributing to the observed activity.

### 3.3. Phenolic and Flavonoid Profiles Determined by HPLC

HPLC-DAD screening indicated peaks corresponding to gallic acid, quercetin, and cinnamic acid based on comparisons of retention times and UV spectra with authentic standards. However, targeted LC–MS/MS supported the assignment of quercetin but did not detect gallic acid or cinnamic acid under the present analytical conditions. Therefore, gallic acid and cinnamic acid should be regarded as HPLC-DAD assignments that were not independently supported by targeted LC–MS/MS, whereas quercetin was consistently detected by both analytical approaches. HPLC analysis demonstrated that wild and in vitro-derived *C. lampangensis* shared several phenolic and flavonoid constituents while also displaying source- and tissue-specific differences.

Quercetin was detected in all tissue and source combinations examined in the present study. This pattern differs from that reported for *K. larsenii*, in which quercetin was reportedly restricted to the pseudostems of wild plants [33]. Such interspecific differences may reflect variation in genotype, tissue differentiation, culture medium, developmental stage, or analytical sensitivity. The comparatively high accumulation of vanillic acid in the in vitro-derived aerial ethyl acetate extract is also consistent with evidence that in vitro conditions can modify phenylpropanoid metabolism. Similar phenolic acids and flavonoids, including gallic acid, vanillic acid, 4-hydroxybenzoic acid, caffeic acid, p-coumaric acid, ferulic acid, rutin, and quercetin, have been reported in *C. longa* rhizomes [37].

The absence of some compounds from particular extracts should not necessarily be interpreted as their complete biological absence. Their concentrations may have been below the analytical detection limit, or the compounds may not have been efficiently recovered by the selected solvent.

### 3.4. LC–MS/MS Analysis

Targeted LC–MS/MS analysis provided complementary evidence for the occurrence of several phenolic and flavonoid constituents in *C. lampangensis*. In particular, p-coumaric acid, 4-hydroxybenzoic acid, chlorogenic acid, and quercetin were consistently detected in all eight extracts, indicating that these compounds were widely distributed across both wild and in vitro-derived materials and across both extraction solvents. Ferulic acid and caffeic acid were also broadly distributed, although ferulic acid was not detected in WAET and caffeic acid was not detected in IVBEA. In contrast, rutin, vanillic acid, and syringic acid showed more restricted distributions, with rutin detected in WAET, IVAET, and IVAEA, vanillic acid detected only in WAEA and IVAEA, and syringic acid detected exclusively in IVAEA.

The detection of several phenolic constituents in the in vitro-derived extracts, particularly IVAET, IVBET, IVAEA, and IVBEA, indicates that tissue-cultured material retained the capacity to produce a range of phenolic and flavonoid metabolites. However, the present LC–MS/MS analysis was designed as a targeted screening approach rather than an absolute quantitative determination. Therefore, the detection patterns should be interpreted primarily as evidence of compound occurrence and distribution rather than as direct comparisons of compound abundance among extracts.

The LC–MS/MS results also showed that gallic acid, cinnamic acid, and catechin were not detected in any of the eight extracts under the analytical conditions used, despite being included as authentic reference standards. This finding indicates that not all compounds previously considered in the HPLC screening were confirmed by targeted LC–MS/MS. Differences between HPLC and LC–MS/MS observations may reflect differences in analytical selectivity, sensitivity, chromatographic behavior, ionization efficiency, or matrix effects. Accordingly, the LC–MS/MS data are best regarded as complementary targeted evidence supporting the phytochemical characterization of *C. lampangensis*, rather than as complete confirmation of all compounds assigned by HPLC.

Overall, the recurrent detection of p-coumaric acid, 4-hydroxybenzoic acid, chlorogenic acid, quercetin, ferulic acid, and caffeic acid across multiple extracts supports the presence of a chemically diverse phenolic profile in both wild and in vitro-derived materials. These findings further support the use of in vitro-derived biomass as renewable material for subsequent phytochemical characterization, compound isolation, and bioactivity-guided investigations.

### 3.5. Volatile and Semi-Volatile Profiles Determined by GC–MS

GC–MS profiling revealed marked qualitative and relative compositional differences among wild and in vitro-derived tissues and between the two extraction solvents. Fatty acid ethyl esters were the predominant group of tentatively identified compounds in the wild belowground and in vitro-derived aerial extracts. These included ethyl palmitate, ethyl linoleate, ethyl oleate, and ethyl stearate. Their combined relative peak areas were 50.92% in WBET, 51.25% in WBEA, 55.57% in IVAET, and 63.31% in IVAEA. Ethyl palmitate was the major constituent in WBET, WBEA, and IVAET, accounting for 19.81%, 25.80%, and 34.14% of the total chromatographic peak area, respectively. In IVAEA, ethyl palmitate was detected at 21.19%, together with relatively high proportions of ethyl linoleate (19.50%) and ethyl oleate (15.73%). Fatty acid ethyl esters have also been reported in other Zingiberaceae taxa, including *Kaempferia grandifolia* [15], *Zingiber zerumbet* [38], and *Z. officinale* [39]. Their variable distribution among the present extracts may be associated with differences in tissue type, developmental stage, lipid metabolism, and solvent affinity.

Camphor was detected exclusively in the ethanol extract of wild belowground tissues at a relative peak area of 1.46%. This tissue-specific occurrence suggests that some volatile constituents may be associated with mature field-grown belowground organs or may occur below the detection limit in the corresponding in vitro-derived tissues. Coumarin was detected in the wild aerial ethanol and ethyl acetate extracts at 0.83% and 1.02%, respectively, and reached its highest relative abundance in the in vitro-derived aerial ethanol extract at 5.75%. Hexadecane, octadecane, and isopropyl myristate were detected in both wild belowground and in vitro-derived aerial extracts, although their relative abundances differed among samples.

### 3.6. Conservation Relevance, Study Limitations, and Future Directions

The previously established in vitro propagation system demonstrated that *C. lampangensis* can be multiplied and acclimatized under controlled conditions. The present study extends that work by showing that in vitro-derived tissues retain detectable phenolic, flavonoid, volatile, and semi-volatile constituents, together with antioxidant and tyrosinase-inhibitory activities. From a conservation perspective, this finding is important because plant cell and tissue culture provides an established approach for the propagation, improvement, and ex situ conservation of medicinal plants [40]. Accordingly, tissue cultured biomass may provide renewable experimental material for phytochemical and biological investigations without continued destructive collection from the species restricted natural population.

Several limitations affect the interpretation of the present study. First, the anatomical composition of the wild and in vitro-derived belowground fractions was not equivalent: the wild fraction included both rhizome and root tissues, whereas the in vitro-derived fraction consisted primarily of roots. Second, the samples differed in developmental stage and growth environment. Plant origin, organ identity, tissue maturity, and culture conditions were therefore partly confounded, and the observed chemical differences cannot be attributed exclusively to in vitro culture or the effects of plant growth regulators. Third, the antioxidant and tyrosinase-inhibition assays were chemical- or enzyme-based in vitro screening methods and therefore do not establish cellular efficacy, bioavailability, safety, or pharmacological activity. Finally, the tentative GC–MS identifications require confirmation using authentic standards or complementary spectroscopic techniques, while the quantitative HPLC data should be verified using appropriate analytical validation parameters.

Future investigations should compare anatomically equivalent tissues collected at matched developmental stages from wild, acclimatized, and greenhouse-grown plants. The stability of phytochemical profiles across successive culture generations should also be evaluated. Bioactivity-guided fractionation, structural confirmation of the active compounds, enzyme-kinetic analysis, cell-based assays, and preliminary safety evaluations will be necessary to determine the biological relevance of the observed activities. Overall, the present findings support in vitro-derived *C. lampangensis* biomass as a potentially renewable and conservation-compatible source of material for further phytochemical and bioactivity-guided research. Nevertheless, additional validation is required before any pharmacological, cosmetic, or commercial applications can be proposed.

## 4. Materials and Methods

### 4.1. Chemicals, Reagents, and Reference Standards

All chemicals and reagents used in this study were of analytical grade unless otherwise specified. The authentic standards comprised ascorbic acid (99%, Sigma-Aldrich, St. Louis, MO, USA), gallic acid (98%, Merck KGaA, Darmstadt, Germany), 4-hydroxybenzoic acid (99%, Sigma-Aldrich, St. Louis, MO, USA), chlorogenic acid (≥95%, Sigma-Aldrich, St. Louis, MO, USA), vanillic acid (97%, Sigma-Aldrich, St. Louis, MO, USA), caffeic acid (≥98.0%, Sigma-Aldrich, St. Louis, MO, USA), syringic acid (≥98.0%, Sigma-Aldrich, St. Louis, MO, USA), p-coumaric acid (≥98.0%, Sigma-Aldrich, St. Louis, MO, USA), ferulic acid (≥99.0%, Sigma-Aldrich, St. Louis, MO, USA), cinnamic acid (99.0%, Sigma-Aldrich, St. Louis, MO, USA), catechin (≥96.0%, Sigma-Aldrich, St. Louis, MO, USA), rutin (95%, Sigma-Aldrich, St. Louis, MO, USA), and quercetin (≥95%, Sigma-Aldrich, St. Louis, MO, USA).

These standards were obtained from Sigma-Aldrich/Merck. Kojic acid (98.5%, HPLC), used as the reference inhibitor in the tyrosinase-inhibitory assay, was purchased from Sigma-Aldrich (St. Louis, MO, USA). The grades and purities of the reference compounds were based on the specifications provided by the manufacturers. HPLC- or LC–MS-grade solvents were used for chromatographic analyses, as appropriate.

### 4.2. Plant Material and Sample Preparation

Wild specimens of *Curcuma lampangensis* Saensouk, Maknoi & Rakarcha were collected from Lampang Province, northern Thailand, in August 2023. The plants were identified and authenticated by Associate Professor Dr. Surapon Saensouk, Walai Rukhavej Botanical Research Institute, Mahasarakham University, Thailand. A voucher specimen (Saensouk P10) was deposited in the Mahasarakham University Herbarium.

Sterile cultures of *C. lampangensis* were established from mature seeds collected from healthy fruits. Only mature, plump, and healthy brown seeds were selected for culture establishment. The seeds were cleaned and surface-sterilized sequentially with 15% and 10% commercial sodium hypochlorite (NaOCl) solutions for 15 and 10 min, respectively, following Phoothonrat et al.’s method [12]. After surface sterilization, the seeds were aseptically cultured on Murashige and Skoog (MS) medium supplemented with 2 mg/L benzyladenine (BA; Sigma–Aldrich, Budapest, Hungary) and 0.5 mg/L 1-naphthaleneacetic acid (NAA; Sd Fine–Chem Limited, Mumbai, India). The medium contained 30 g/L sucrose, was adjusted to pH 5.7–5.8, and was solidified with 7 g/L agar, followed by autoclaving at 121 °C for 20 min. Cultures were maintained at 25 ± 2 °C under a 16 h photoperiod with fluorescent illumination of 27 µmol m^−2^ s^−1^, and were subcultured onto the same medium every 8 weeks to multiply the plantlets. In vitro-derived plant materials used for phytochemical analyses and biological activity assays were collected at the end of the 8-week culture period. At this culture endpoint, each explant produced approximately four shoots with a mean shoot length of 4.0 cm and approximately 10 roots with a mean root length of 2.5 cm.

**Figure 2:**
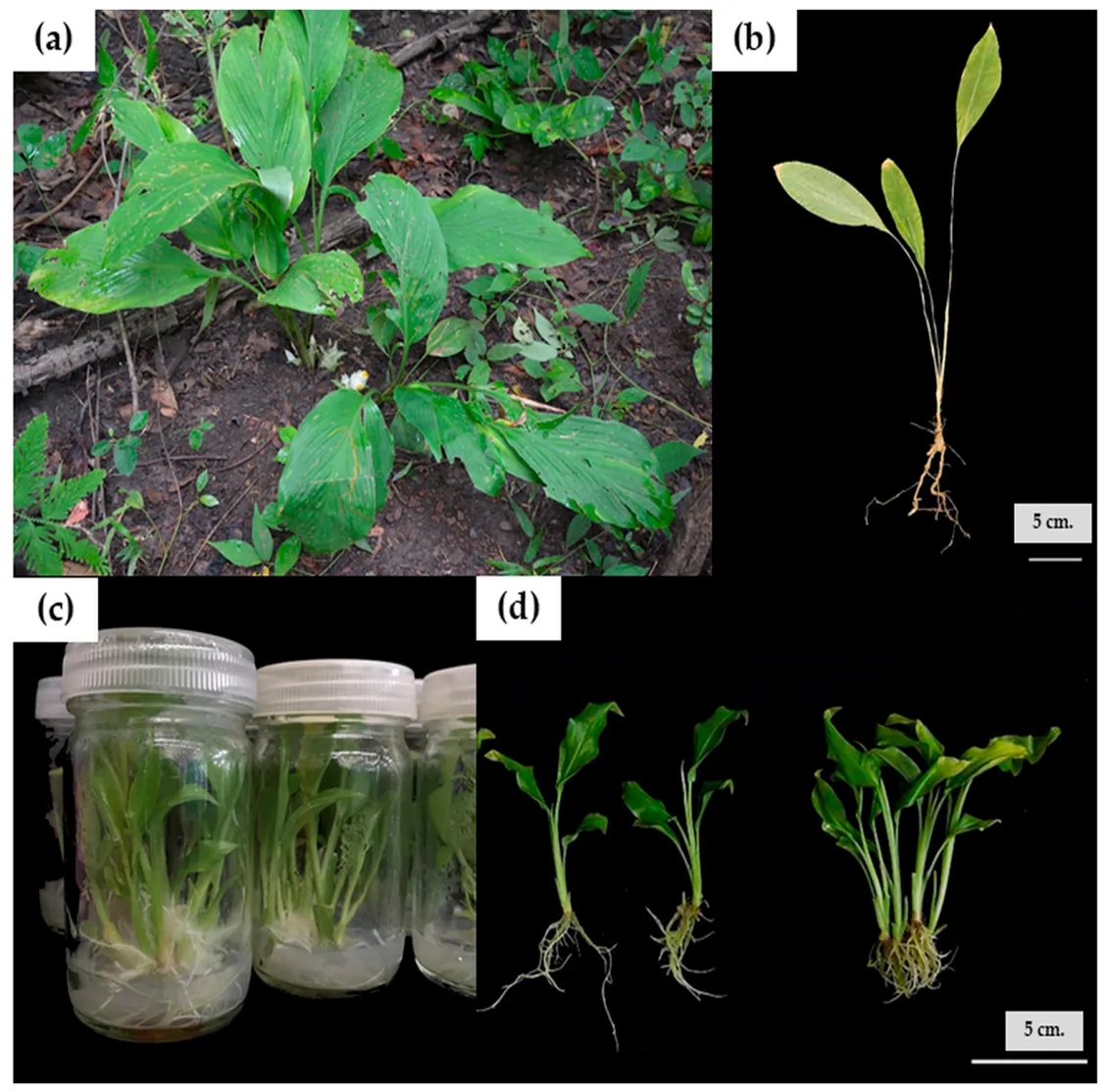
Morphological characteristics of *C. lampangensis* and plant materials used in this study. (**a**) Wild plant growing in its natural habitat; (**b**) representative whole wild plant showing aerial and belowground parts; (**c**) in vitro cultures of *C. lampangensis*; and (**d**) in vitro-derived plantlets showing shoot and root development. Scale bars = 5 cm in (**b**,**d**).

The wild plant material was separated into aerial tissues (WA), consisting of leaves and pseudostems, and belowground tissues (WB), consisting of rhizomes and roots. For comparison, in vitro-derived plantlets were cultured on Murashige and Skoog medium supplemented with 2 mg/L 6-benzyladenine (BA) and 0.5 mg/L α-naphthaleneacetic acid (NAA). The in vitro-derived plantlets were separated into aerial tissues (IVA), comprising leaves and pseudostems, and belowground tissues (IVB), comprising roots (Figure 2).

The wild and in vitro-derived samples were washed thoroughly with running tap water and subsequently rinsed with distilled water. The samples were stored at −20 °C before drying in a forced-air oven at 50 °C for 48 h or until a constant weight was obtained. The dried materials were ground to a homogeneous powder and stored in airtight containers at 4 °C until extraction. Each biological replicate consisted of material obtained from three independently collected or cultured plants.

#### 4.2.1. Extraction for Colorimetric and Biological Assays

A finely powdered plant sample (0.5 g) was used as the starting material for extraction, which was carried out separately with 20 mL of either ethanol or ethyl acetate. The sample-solvent mixtures were placed in an incubator shaker set at 37 °C and agitated at a constant speed of 120 rpm for 24 h to facilitate maximal dissolution of bioactive compounds, followed by sonication in an ultrasonic bath (Elmasonic Select 60, Elma Schmidbauer GmbH, Singen, Germany) at 37 kHz for 30 min. The mixtures were then filtered through Whatman No. 1 filter paper, and the filtrates were centrifuged at 4000 rpm (2147× *g*) for 30 min at room temperature. The resulting supernatants were carefully collected, adjusted to a final volume of 20 mL with the respective solvent, and stored at −20 °C to preserve their integrity for subsequent phytochemical and antioxidant activity analyses.

#### 4.2.2. Sample Preparation for HPLC-DAD, LC–MS/MS, and GC–MS Analyses

Aliquots of the extracts prepared as described above were filtered through a 0.22 µm nylon membrane filter to remove particulate matter prior to HPLC-DAD, LC–MS/MS, and GC–MS analyses.

### 4.3. Determination of Total Phenolic Content

Total phenolic content was determined using the Folin–Ciocalteu method modified from Sari et al. [41]. A 20 µL aliquot of the appropriately diluted extract or gallic acid standard was mixed with 20 µL of 10% (*v*/*v*) Folin–Ciocalteu reagent in a 96-well microplate. After mixing for 1 min and incubation for 5 min at room temperature, 200 µL of 7% (*w*/*v*) sodium carbonate solution and 10 µL of deionized water were added. The reaction mixture was incubated in the dark at 25 °C for 120 min, after which the absorbance was measured at 750 nm using a microplate reader.

Gallic acid was used as the standard reference compound for quantification, with calibration curves prepared at concentrations of 15, 45, 75, 105, and 135 µg/mL. The total phenolic content of the plant samples was subsequently expressed as milligrams of gallic acid equivalents (GAE) per gram of dry weight (DW).

### 4.4. Determination of Total Flavonoid Content

Total flavonoid content was determined using the aluminum chloride colorimetric method modified from Sari et al. [41]. A 50 µL aliquot of the appropriately diluted extract or quercetin standard was mixed with 100 µL of 95% methanol and 20 µL of 10% (*w*/*v*) aluminium chloride solution. After incubation for 3 min at room temperature, 20 µL of 1 M potassium acetate and 60 µL of methanol were added. The reaction mixture was incubated in the dark at room temperature for 40 min, and absorbance was measured at 430 nm.

Quercetin standards were prepared in ethanol at concentrations of 5, 25, 45, 65, and 85 µg/mL, with methanol serving as the reagent blank. The total flavonoid content (TFC) was calculated based on the quercetin calibration curve and expressed as milligrams of quercetin equivalents per gram of dry weight (mg QE/g DW).

### 4.5. DPPH Radical-Scavenging Assay

DPPH radical-scavenging activity was evaluated using a method modified from Lopes et al. [42]. A 150 µmol/L DPPH solution was freshly prepared in 99.5% ethanol. A 66 µL aliquot of extract, ascorbic acid standard, or solvent control was mixed with 134 µL of DPPH solution in a 96-well microplate. The mixture was incubated in the dark at room temperature for 45 min, and absorbance was measured at 517 nm. The absorbance values of the control (Ac) and samples (As) were measured. Ascorbic acid was prepared at concentrations of 1.95–125 µg/mL. The percentage of radical scavenging activity for each sample was calculated using the following equation:

Percentage radical scavenging = [(Ac − As)/Ac] × 100.

Extracts that possessed higher radical scavenging activity than 50% by DPPH assay were further evaluated for IC_50_ values.

### 4.6. ABTS Radical-Scavenging Assay

ABTS radical-scavenging activity was determined according to Chumroenphat et al. [37]. The ABTS radical cation was generated by mixing 7.4 mmol/L ABTS with 2.6 mmol/L potassium persulfate at a 1:1 (*v*/*v*) ratio and incubating the mixture in the dark at room temperature for 12–16 h. Before use, the solution was diluted with 95% ethanol to an absorbance of (0.70 ± 0.02) at 734 nm.

A 190 µL aliquot of the diluted ABTS solution was mixed with 10 µL of extract, ascorbic acid standard, or solvent control. After incubation in the dark for 120 min, absorbance was measured at 734 nm. Ascorbic acid standards were prepared at 15–165 µg/mL. ABTS inhibition was calculated using the background-corrected equation described for the DPPH assay.

The absorbance values of the control (Ac) and samples (As) were measured. The percentage of radical scavenging activity for each sample was calculated using the following equation:

Percentage radical scavenging = [(Ac − As)/Ac] × 100.

Extracts that possessed higher radical scavenging activity than 50% by ABTS assay were further evaluated for IC_50_ values.

### 4.7. Tyrosinase-Inhibitory Activity

Tyrosinase-inhibitory activity was evaluated using mushroom tyrosinase and L-DOPA as the substrate, following a method modified from Karkouch et al. [43]. Mushroom tyrosinase solution (100 U/mL) and L-DOPA solution (2.5 mM) were prepared in sodium phosphate buffer (0.1 mol/L; pH 6.8).

For the sample reaction, 10 µL of extract was mixed with 40 µL of sodium phosphate buffer and 70 µL of tyrosinase solution. After preincubation at room temperature for 10 min, 80 µL of L-DOPA solution was added. The reaction was incubated for a further 10 min, and absorbance was measured at 475 nm.

A was the enzyme control, B was the corresponding blank without enzyme, C was the sample reaction containing extract and enzyme, and D was the sample blank without enzyme. Tyrosinase inhibition was calculated as:

% Inhibition = 100 [(A − B) − (C − D)]/(A − B)

Kojic acid was used as the positive control. Extracts exhibiting greater than 50% tyrosinase inhibitory activity during initial screening were further evaluated to determine their IC_50_ values.

To further evaluate the potential contribution of the phenolic compounds identified by HPLC to the observed tyrosinase-inhibitory activity, the 12 authentic standards used for HPLC identification—gallic acid, 4-hydroxybenzoic acid, chlorogenic acid, vanillic acid, caffeic acid, syringic acid, p-coumaric acid, ferulic acid, cinnamic acid, catechin, rutin, and quercetin—were individually evaluated using the same mushroom tyrosinase assay. Kojic acid was included as a reference tyrosinase inhibitor. Each standard and kojic acid was initially tested at a final concentration of 100 µM. Compounds exhibiting ≥50% tyrosinase inhibition at this concentration were subsequently evaluated over a range of concentrations to determine their half-maximal inhibitory concentration (IC_50_) values. Appropriate controls and blanks were prepared in parallel, and all measurements were performed in triplicate.

### 4.8. HPLC Analysis of Phenolic Acids and Flavonoids

Phenolic acids and flavonoids were analyzed using an HPLC system equipped with a diode-array detector (GL Sciences Inc., Tokyo, Japan). Separation was performed on an Inertsil ODS-3 C18 column (250 mm × 4.6 mm, 5 µm) maintained at 38 °C, using the gradient elution conditions described by Kubola and Siriamornpun [44]. The mobile phase consisted of purified water acidified with acetic acid to pH 2.74 (solvent A) and acetonitrile (solvent B), delivered at 0.8 mL min^−1^. The gradient program was 5–9% B (0–5 min), 9% B (5–15 min), 9–11% B (15–22 min), 11–18% B (22–38 min), 18–23% B (38–43 min), 23–90% B (43–44 min), 90–80% B (44–45 min), and 80% B (45–55 min), followed by a decrease to 5% B from 55 to 60 min. The column was re-equilibrated at 5% B for 5 min before the next injection. The injection volume was 20 µL, and detection was carried out at 280 nm for phenolic acids and catechin and at 370 nm for flavonoids. Compounds were identified by comparing their retention times and UV spectra with those of authentic standards and quantified using the external-standard method. The analytical standards were selected to represent phenolic acids and flavonoids relevant to the phytochemical characterization of *Curcuma* materials and to permit comparison of the chemical profiles of wild and in vitro-derived tissues using the adopted HPLC-DAD method.

### 4.9. LC–MS/MS Conditions for Phenolic and Flavonoid Analysis

Phenolic and flavonoid compounds were analyzed using the gradient elution conditions described by Myrtsi et al. [45], with slight modifications, on a Shimadzu LCMS-8030 triple-quadrupole mass spectrometer (Shimadzu Corporation, Kyoto, Japan) equipped with an electrospray ionization (ESI) source operating in negative-ion mode. Chromatographic separation was achieved using an InertSustain^®^ C18 column (150 × 2.1 mm, 3 µm) maintained at 30 °C. The mobile phase consisted of 0.1% (*v*/*v*) formic acid in ultrapure water (mobile phase A) and LC–MS-grade acetonitrile (mobile phase B), delivered at a flow rate of 0.30 mL/min. The injection volume was 1 µL. The gradient program was as follows: 5% B at 2.0 min, increased to 95% B at 6.5 min, maintained at 95% B until 9.0 min, and returned to 5% B at 10.0 min for column re-equilibration. The total analytical run time was 17 min.

Mass spectrometric detection was performed in multiple reaction monitoring (MRM) mode. The ESI gas flow rate was set at 2.3 L/min, with a desolvation line (DL) temperature of 250 °C and a heat-block temperature of 400 °C. The same 12 authentic reference standards used for HPLC-DAD profiling were used for targeted LC–MS/MS identification. Compound occurrence was assessed by agreement of retention time and compound-specific MRM transition(s) with authentic standards analyzed under identical conditions. The LC–MS/MS analysis was used as a targeted confirmation screen; therefore, the results are reported qualitatively as detected or not detected.

### 4.10. GC–MS Analysis

GC–MS analysis was performed according to a previously described method [15], with modifications, using an Agilent 8890 GC system coupled to an Agilent 5977C mass-selective detector (Agilent Technologies, Santa Clara, CA, USA). Chromatographic separation was achieved on a DB-5MS + DG capillary column (40 m × 0.25 mm i.d., 0.25 µm film thickness), with helium used as the carrier gas at a constant flow rate of 1.0 mL min^−1^. A 1 µL aliquot of each sample was injected in splitless mode at an injector temperature of 250 °C. The oven temperature was initially maintained at 70 °C and then increased to 280 °C at a rate of 5 °C min^−1^, with a total run time of 53 min. Mass spectra were acquired in electron-ionization mode at 70 eV over an *m*/*z* range of 40–550 using full-scan acquisition. The transfer-line, ion-source, and quadrupole temperatures were maintained at 280, 230, and 150 °C, respectively, and a solvent delay of 3 min was applied. Compounds were tentatively identified by comparing their mass spectra with those in the NIST and Wiley mass spectral libraries. Relative abundances were calculated by peak-area normalization and expressed as percentages of the total chromatographic peak area.

### 4.11. Statistical Analysis

Data are presented as means ± SD of three independent biological replicates. Normality and homogeneity of variance were evaluated using the Shapiro–Wilk and Levene’s tests, respectively. Differences among the eight extract preparations were assessed by one-way analysis of variance (ANOVA) followed by Tukey’s honestly significant difference (HSD) post hoc test, with statistical significance set at *p* < 0.05.

## 5. Conclusions

This study extends previous in vitro propagation research on *C. lampangensis* by demonstrating that in vitro-derived tissues retain measurable phytochemical constituents and biological activities. Wild belowground extracts exhibited comparatively high total phenolic content and DPPH radical-scavenging activity, whereas the ethyl acetate extract of in vitro-derived aerial tissues showed the highest total flavonoid content and ABTS radical-scavenging activity. The ethyl acetate extract of in vitro-derived belowground tissues exhibited the strongest tyrosinase-inhibitory activity among the tested extracts. HPLC-DAD, targeted LC–MS/MS, and GC–MS analyses further revealed distinct chemical profiles associated with plant origin, tissue type, and extraction solvent.

Although the in vitro-derived and wild-collected materials were not anatomically or chemically equivalent, the cultured tissues retained phytochemical constituents and biological activities relevant to further investigation. The observed variation should therefore be interpreted as reflecting the combined influence of tissue composition, developmental status, growth environment, and extraction solvent rather than in vitro culture alone. Overall, these findings link in vitro propagation with biochemical characterization and support in vitro-derived biomass as a potentially renewable, conservation-compatible research material for this critically endangered Thai endemic species. Further studies using anatomically comparable tissues, rigorous compound identification, and bioactivity-guided fractionation are warranted to clarify the chemical stability and biological relevance of cultured biomass.

## Figures and Tables

**Table 1:** Total phenolic content and total flavonoid content of *C. lampangensis* extracts.

Plant Extracts	TPC (mg GAE/g DW)	TFC (mg QE/g DW)
WAET	1.30 ± 0.02 ^f^	0.41 ± 0.00 ^ef^
IVAET	1.94 ± 0.02 ^d^	1.12 ± 0.02 ^c^
WBET	4.05 ± 0.02 ^b^	0.91 ± 0.00 ^d^
IVBET	1.44 ± 0.01 ^e^	0.36 ± 0.01 ^f^
WAEA	0.75 ± 0.01 ^h^	0.43 ± 0.01 ^e^
IVAEA	2.61 ± 0.01 ^c^	1.59 ± 0.04 ^a^
WBEA	5.21 ± 0.01 ^a^	1.19 ± 0.02 ^b^
IVBEA	1.14 ± 0.05 ^g^	0.27 ± 0.00 ^g^

Values are presented as means ± SD of three independent biological replicates. Different lowercase letters within the same column indicate significant differences among extracts according to one-way ANOVA followed by Tukey’s honestly significant difference test (*p* < 0.05). TPC, total phenolic content; TFC, total flavonoid content; GAE, gallic acid equivalents; QE, quercetin equivalents; DW, plant dry weight; WAET, wild aerial ethanol extract; IVAET, in vitro-derived aerial ethanol extract; WBET, wild belowground ethanol extract; IVBET, in vitro-derived belowground ethanol extract; WAEA, wild aerial ethyl acetate extract; IVAEA, in vitro-derived aerial ethyl acetate extract; WBEA, wild belowground ethyl acetate extract; IVBEA, in vitro-derived belowground ethyl acetate extract.

**Table 2:** Antioxidant and tyrosinase inhibitory activities of wild and in vitro-derived *C. lampangensis* extracts.

Plant Extracts	DPPH Assay % Inhibition	ABTS Assay % Inhibition	Tyrosinase % Inhibition
WAET	30.41 ± 0.25 ^d^	23.00 ± 0.4 ^e^	44.33 ± 1.70 ^bc^
IVAET	35.89 ± 0.47 ^c^	45.56 ± 0.49 ^c^	43.34 ± 0.75 ^b^
WBET	70.48 ± 0.25 ^a^	59.46 ± 0.57 ^b^	43.91 ± 0.57 ^b^
IVBET	24.86 ± 0.30 ^e^	21.04 ± 0.41 ^f^	42.49 ± 3.27 ^b^
WAEA	16.58 ± 0.41 ^h^	19.84 ± 0.48 ^f^	44.19 ± 1.50 ^bc^
IVAEA	19.04 ± 0.86 ^g^	63.11 ± 0.38 ^a^	43.63 ± 0.49 ^b^
WBEA	42.53 ± 0.38 ^b^	46.59 ± 0.11 ^c^	49.29 ± 1.72 ^ab^
IVBEA	20.75 ± 0.49 ^f^	25.18 ± 0.11 ^d^	52.12 ± 1.23 ^a^

Values are presented as means ± SD of three independent biological replicates. Different lowercase letters within the same column indicate significant differences among extracts according to one-way ANOVA followed by Tukey’s honestly significant difference test (*p* < 0.05).

**Table 3:** LC–MS/MS identification parameters and distribution of targeted phenolic and flavonoid compounds in *C. lampangensis* extracts.

Compound	MRM Transition (*m*/*z*)	CE (eV)	RT (min)	Detected Extract(s)
Gallic acid	169.00 → 125.00 (79.00)	17.4 (26.7)	5.695	ND
*p*-Coumaric acid	163.00 → 119.00 (93.00)	17.8 (33.6)	7.316	WAET, IVAET, WBET, IVBET, WAEA, IVAEA, WBEA, IVBEA
Ferulic acid	193.05 → 134.00 (178.05)	18.4 (15.8)	7.403	WBET, IVBET, WAEA, IVAEA, WBEA, IVBEA, IVAET
Cinnamic acid	147.05 → 103.00 (77.00)	16.8 (26.1)	8.170	ND
Quercetin	301.05 → 151.00 (179.00)	24.1 (19.0)	7.802	WAET, IVAET, WBET, IVBET, WAEA, IVAEA, WBEA, IVBEA
4-Hydroxybenzoic acid	136.95 → 93.00 (65.00)	18.4 (35.6)	7.034	WAET, IVAET, WBET, IVBET, WAEA, IVAEA, WBEA, IVBEA
Chlorogenic acid	353.15 → 191.00	18.4	6.722	WAET, IVAET, WBET, IVBET, WAEA, IVAEA, WBEA, IVBEA
Vanillic acid	167.05 → 152.00 (108.00)	19.4 (19.4)	7.138	WAEA, IVAEA
Caffeic acid	179.05 → 135.00 (89.00)	17.8 (34.6)	7.119	WAET, IVAET, WBET, IVBET, WAEA, IVAEA, WBEA
Syringic acid	197.05 → 182.00 (122.90)	17.6 (26.3)	7.170	IVAEA
Catechin	289.10 → 245.10 (203.00)	15.2 (20.0)	6.912	ND
Rutin	609.10 → 300.00 (271.00)	38.3 (58.0)	6.995	WAET, IVAET, IVAEA

MRM, multiple reaction monitoring; CE, collision energy; RT, retention time; ND, not detected. Values in parentheses represent qualifier product ions and their corresponding collision energies. All analytes were monitored in negative electrospray ionization mode. Compound identification was based on agreement of retention times and MRM transitions with authentic reference standards analyzed under identical LC–MS/MS conditions.

**Table 4:** Tentatively identified constituents in wild and in vitro-derived *C. lampangensis* extracts analyzed by GC–MS.

No	Name of the Compound	RT	Peak Area %							
			Wild				In Vitro-Derived			
			WAET	WAEA	WBET	WBEA	IVAET	IVAEA	IVBET	IVBEA
1	Camphor	12.244	ND	ND	1.46	ND	ND	ND	ND	ND
2	Coumarin	20.340	0.83	1.02	ND	ND	5.75	ND	ND	ND
3	Hexadecane	23.027	ND	ND	4.83	4.66	4.41	2.12	ND	ND
4	Octadecane	27.463	ND	ND	5.41	6.90	4.94	5.85	ND	ND
5	Isopropyl myristate	28.046	ND	ND	4.55	5.08	4.06	4.82	ND	ND
6	Hexadecanoic acid, ethyl ester (Ethyl palmitate)	31.397	0.98	0.54	19.81	25.80	34.14	21.19	ND	ND
7	1-Naphthalenepropanol, α-ethenyldecahydro-α,5,5,8a-tetramethyl-2-methylene-, [1S-[1α(S),4aβ,8aα]] (Manool)	34.235	ND	ND	ND	ND	ND	ND	ND	36.40
8	Linoleic acid ethyl ester (Ethyl linoleate)	34.469	ND	ND	12.71	15.09	19.54	19.50	ND	ND
9	Ethyl Oleate	34.714	ND	ND	9.59	2.89	1.89	15.73	ND	ND
10	Octadecanoic acid, ethyl ester (Ethyl stearate)	35.092	ND	ND	8.81	7.47	ND	6.89	ND	ND

## Data Availability

The data presented in this study are available on request from the corresponding author. The data are not publicly available due to institutional data policy and ongoing research.
